# EZH2 blockade reverses doxorubicin resistance by inducing metabolic vulnerability and enhancing DNA damage in breast cancer

**DOI:** 10.3389/fphar.2026.1786648

**Published:** 2026-05-14

**Authors:** Xiaomin Wang, Yuhang Ding, Yunxiao Mai, Ruinan Li, Xinyu Shao, Wenlong Chen, Yiming Li, Luhaoxiang Liu, Haoran Wang, Kangkang Liu, Yuanjie Niu, Jianmin Li, Guoping Xu, Yang Zhao

**Affiliations:** 1 Department of Radiology, The Second Hospital of Tianjin Medical University, Tianjin Medical University, Tianjin, China; 2 Tianjin Institute of Urology, The Second Hospital of Tianjin Medical University, Tianjin Medical University, Tianjin, China; 3 National Clinical Research Center for Geriatric Disorders, Xiangya Hospital, Central South University, Changsha, China; 4 Department of Breast Surgery, Xiangya Hospital, Central South University, Changsha, China; 5 Haihe Laboratory of Synthetic Biology, Tianjin, China

**Keywords:** breast cancer, DNA damage, doxorubicin, EZH2, metabolic vulnerability, resistance

## Abstract

**Background:**

Doxorubicin (DOX) resistance remains a major obstacle to effective chemotherapy in breast cancer. However, the pharmacologically actionable regulators sustaining this resistant phenotype and its therapeutic vulnerabilities remain incompletely defined.

**Methods:**

DOX-resistant breast cancer cell models were established and treated with the EZH2 inhibitors tazemetostat or GSK126, alone or in combination with DOX. Cell viability, oxidative stress, DNA damage, and mitochondrial function were assessed *in vitro*. Transcriptomic profiling was performed to identify pathway alterations. A pH-responsive liposomal system for delivery of DOX and tazemetostat was developed and evaluated *in vivo*.

**Results:**

EZH2 was highly expressed in breast cancer and correlated with poor clinical outcomes. DOX treatment induced adaptive upregulation of EZH2 in both sensitive and resistant cells. Pharmacological inhibition of EZH2 markedly restored DOX sensitivity and exhibited strong synergistic cytotoxicity in resistant models. EZH2 blockade enhanced DOX-induced oxidative stress and DNA damage, with concomitant suppression of multiple DNA repair pathways and increased γH2AX accumulation. Transcriptomic and functional analyses revealed disrupted mitochondrial function and energy metabolism, characterized by loss of mitochondrial membrane potential and ATP depletion. *In vivo*, combined EZH2 inhibition and DOX significantly suppressed tumor growth, while pH-responsive liposomal delivery further enhanced antitumor efficacy and reduced systemic toxicity.

**Conclusion:**

EZH2 is a critical determinant of DOX resistance in breast cancer by sustaining DNA damage tolerance and metabolic homeostasis. Pharmacological targeting of EZH2 in combination with DOX represents a rational strategy to overcome chemoresistance in breast cancer.

## 1 Introduction

Breast cancer (BRCA) remains a leading malignancy among women worldwide (Bray et al., 2024). Despite advances in targeted therapies and immunotherapy, cytotoxic chemotherapy, particularly anthracyclines such as doxorubicin (DOX), remains the cornerstone of systemic treatment across stages and molecular subtypes (Harbeck and Gnant, 2016; Bisht et al., 2024). DOX exerts antitumor effects mainly through DNA intercalation/topoisomerase II inhibition, which generates double-strand breaks, and by triggering the accumulation of reactive oxygen species (ROS) that induce lethal oxidative stress (Agudelo et al., 2014). However, acquired DOX resistance is common and drives treatment failure and relapse (Zhang et al., 2025; Mattioli et al., 2023). The mechanisms underlying this resistance are multifaceted, including enhanced drug efflux, upregulated DNA damage repair capacity, attenuation of oxidative stress responses, and activation of pro-survival signaling pathways (Doshmanziari et al., 2024; Sanati and Ghafouri-Fard, 2024; Zhang et al., 2025; Wang X. et al., 2024). However, the core molecular programs that can be sustained and therapeutically targeted to reverse DOX resistance remain poorly understood, highlighting the urgent need for novel strategies to overcome chemoresistance and improve patient outcomes.

Increasing evidence indicates that chemotherapy resistance is driven by coordinated epigenetic and metabolic reprogramming rather than isolated molecular events (Xu et al., 2023; Liu et al., 2024). Under therapeutic stress, cancer cells dynamically rewire transcriptional programs while simultaneously adapting their metabolic states to sustain survival (Pan et al., 2025). This integrated response suggests the presence of central regulatory nodes that couple gene expression with metabolic adaptation, thereby maintaining the resistant phenotype and providing potential targets for therapeutic intervention.

Enhancer of zeste homolog 2 (EZH2), the catalytic subunit of PRC2, has emerged as an oncogenic driver and a therapeutic target in multiple malignancies (Duan et al., 2020; Kim and Roberts, 2016). In breast cancer, EZH2 is frequently overexpressed, and has been implicated in promoting aggressive tumor behavior, metastatic progression, and adverse clinical outcomes (Zhang et al., 2022; Kleer et al., 2003). Selective EZH2 inhibitors (EZH2i) have entered clinical practice for certain lymphomas and are under active investigation in solid tumors (Song et al., 2022). However, their clinical benefits remain unsatisfactory and limited to certain hematological malignancies (Italiano et al., 2018), as also highlighted in recent comprehensive reviews (Chen Y. et al., 2024). This may be attributed to the fact that current EZH2i predominantly targets EZH2 methyltransferase activity without effectively countering its non-catalytic oncogenic functions (Wang et al., 2022). Thus, there is a compelling rationale for developing combination regimens that can extend the therapeutic utility of EZH2 inhibition in solid tumors including BRCA.

EZH2 has emerged as a central regulator of therapeutic resistance across multiple treatment modalities, including chemotherapy, endocrine therapy, and targeted therapy (Chen Y. et al., 2024; Bao et al., 2020; Wu et al., 2025; Kaur et al., 2024; Porazzi et al., 2025; Deng et al., 2025; Zhang et al., 2024). Mechanistically, EZH2 coordinates diverse adaptive processes, including suppression of DNA damage response, activation of pro-survival signaling, and epigenetic and metabolic reprogramming under therapeutic stress (Kaur et al., 2024; Fan et al., 2014; Gardner et al., 2017; Hirukawa et al., 2018). In breast cancer, EZH2 contributes to endocrine resistance via epigenetic regulation of genes such as HOXC10 and is linked to increased locoregional recurrence after radiotherapy (Pathiraja et al., 2014; Debeb et al., 2014). Nevertheless, it is still unclear whether EZH2i can re-sensitize DOX-resistant BRCA cells, how they modulate the DNA damage response, and whether they induce additional vulnerabilities, such as mitochondrial or metabolic dysfunction, that could be exploited therapeutically.

In this study, we hypothesized that EZH2 inhibition could overcome DOX resistance in BRCA cells by disrupting the molecular machinery that sustains the resistant phenotype. We employed pharmacological inhibition to systematically investigate the role of EZH2 in DOX resistance in BRCA and explored its potential mechanisms. In DOX-resistant BRCA models, combining EZH2i (tazemetostat (TAZ) and GSK126) with DOX markedly enhanced antitumor efficacy compared with DOX monotherapy. Mechanistically, this effect was mediated by a dramatic increase in intracellular ROS levels, leading to exacerbated oxidative stress. Furthermore, the DNA damage repair pathways were significantly suppressed by the combination strategy, thereby amplifying DOX-induced DNA damage. Moreover, our findings revealed critical energy metabolic vulnerability triggered by EZH2 inhibition. Collectively, these findings support EZH2 as a therapeutic target for overcoming DOX resistance in BRCA and provide a mechanistic rationale for integrating EZH2 inhibition into DOX-based chemotherapy regimens.

## 2 Materials and methods

### 2.1 Materials

Materials used in this study were listed in Supplementary Table S1.

### 2.2 Cell culture

Cell lines were obtained from multiple sources. The DOX-resistant 231-ADR cell line and its parental counterpart (231-WT) were kindly provided by the Feng laboratory at Xiangya Hospital, Central South University and have been previously described and functionally validated. (Zhang et al., 2025; Chen et al., 2022). The MCF7-ADR and its parental MCF7-WT cells were obtained from Liu laboratory (Wang et al., 2023). Cells were maintained in DMEM containing 10% FBS and 1% penicillin–streptomycin at 37 °C in 5% CO_2_.

### 2.3 Evaluation of intracellular ROS

The cells were divided into four experimental groups: control, TAZ, DOX, and TAZ + DOX. For the TAZ and TAZ + DOX groups, the cells were pretreated with 10 µM TAZ for 72 h before the addition of DOX. ROS were monitored by loading cells with 10 µM DCFH-DA or 5 µM DHE (37 °C, 30 min). Nuclei were counterstained with Hoechst (5 μg/mL, 10 min) prior to fluorescence imaging. Fluorescence images were acquired under identical settings, and mean fluorescence intensity was quantified using ImageJ (NIH, United States). Data were obtained from three independent biological replicates (n = 3), with representative images shown.

### 2.4 *In Vitro* cytotoxicity study

Cells were pretreated with or without EZH2i (TAZ or GSK126) for 72 h and then exposed to varying concentrations of DOX. After co-incubation for 24, 48, or 72 h, cell viability was quantified via CCK-8 assay. Each experiment was performed independently four times (n = 4 biological replicates). Cell viability values were calculated relative to the untreated control.

### 2.5 Live/Dead cell staining analysis

After 72 h pre-treatment with or without EZH2i (TAZ or GSK126), cells were treated with DOX for 24 h. Cell survival was evaluated using a Calcein-AM/PI kit and visualized by GLSM. The proportions of live and dead cells were quantified by fluorescence intensity using ImageJ (NIH, United States). Data were obtained from three independent biological replicates, with representative images shown.

### 2.6 qRT-PCR

Total RNA was isolated using TRIzol Reagent. qRT–PCR was performed in triplicate on a CFX Opus 96 system, using the recommended cycling protocol. β-actin served as the reference gene, and relative mRNA levels were calculated using the 2^^(−ΔΔCt)^ method. Primers were supplied by Sangon Biotech (Shanghai, China) and are listed in Supplementary Table S2.

### 2.7 Western blot

Proteins were extracted using RIPA buffer, quantified by BCA assay, separated by SDS–PAGE, and transferred to PVDF membranes. After blocking, membranes were incubated with primary and HRP-conjugated secondary antibodies, and bands were visualized using chemiluminescence. β-Actin served as the loading control. Band intensities were quantified using ImageJ software (NIH, United States) and normalized to β-actin. Each experiment was performed independently three times, and representative blots are shown.

### 2.8 Cell immunofluorescence staining

Cells were fixed, blocked with BSA, and incubated with primary antibodies (EZH2, γH2AX) overnight, followed by fluorophore-conjugated secondary antibodies. Nuclei were counterstained with DAPI (RT, 10 min). Images were captured using GLSM. Fluorescence signals were quantified as described above. Each experiment was performed independently three times (n = 3 biological replicates), and representative images are shown.

### 2.9 Colony formation assay

Cells were seeded and cultured for 2 weeks. Following indicated treatments, colonies were fixed, stained with crystal violet, and counted. Each experiment was performed independently three times (n = 3 biological replicates), and representative images are shown.

### 2.10 Synergy treatment analysis

Drug synergy was assessed in 231-ADR and MCF7-ADR cells. Briefly, cells were seeded in 96-well plates and pretreated with serial dilutions of EZH2i for 72 h. DOX was then added at gradients, followed by 48 h co-incubation. Cell viability was quantified using CCK-8 assay. The drug interaction was analyzed for synergy using the SynergyFinder web tool (https://synergyfinder.org/), based on multiple reference models, including ZIP, Bliss, Loewe, and HSA. The HSA model was used as the primary metric for synergy evaluation. Full drug–response matrices were used for analysis to enable systematic assessment of combinatorial effects across concentration gradients.

### 2.11 Bioinformatics analysis

Public datasets from TCGA and GTEx were used for bioinformatic analyses. Bioinformatics processing was facilitated using the Sangerbox platform (http://sangerbox.com/) or the Xiantao tool (https://xiantaozi.com/). Patient survival analysis was conducted, and Kaplan-Meier curves were generated using the KM plot online tool (https://kmplot.com).

### 2.12 RNA-seq

Transcriptome sequencing was performed by OE Biotech Co. Ltd. Total RNA quality was assessed prior to library construction, and qualified samples were used for cDNA library preparation and sequencing on an Illumina platform. Clean reads were obtained after quality control and aligned to the human reference genome. The gene expression profiles across the four experimental groups (Ctrl, TAZ, DOX, and TAZ + DOX) were first analyzed by PCA, followed by differential expression analysis with DESeq2 (Q < 0.05, |log_2_FC| > 1) and visualization via volcano plots. The identified DEGs were hierarchically clustered and functionally annotated by GO and KEGG enrichment analyses. GSEA was performed using the GSEA computational framework. Trend analysis across Ctrl, DOX, and TAZ + DOX groups was conducted using the Short Time-series Expression Miner (STEM) software.

### 2.13 Mitochondrial membrane potential assay

Mitochondrial membrane potential was assessed using a JC-1 staining kit according to the manufacturer’s instructions. 231-ADR cells were treated with DMSO, TAZ, DOX, or TAZ + DOX, followed by JC-1 staining. Fluorescence images were acquired, and the red/green fluorescence ratio was quantified using ImageJ.

### 2.14 ATP measurement

Intracellular ATP levels were measured using a commercial ATP assay kit. Luminescence was detected using a microplate reader and normalized to protein concentration.

### 2.15 SOD activity assay

SOD activity was determined using a commercial assay kit. Absorbance was measured using a microplate reader, and enzyme activity was calculated accordingly.

### 2.16 Synthesis and characterization of liposomes

Liposomes were prepared by thin-film hydration. Lecithin, cholesteryl hemisuccinate, and DSPE-PEG2000 were dissolved in chloroform, evaporated to form a dry lipid film, and hydrated using an aqueous buffer. TAZ-Lip was obtained by adding TAZ dissolved in N, N-dimethylformamide to the organic phase before film formation, whereas DOX-Lip was obtained by adding DOX during hydration. The suspensions were sonicated, extruded through 100 nm membranes, and purified by ultrafiltration.

The particle size, PDI, and zeta potential were measured by DLS, and the morphology was examined by TEM. The drug-loading efficiency was determined by HPLC (TAZ) or UV-Vis (DOX).

### 2.17 *In vivo* antitumor efficacy assessment

BALB/c nude mice bearing 231-ADR xenografts were randomized into five groups (n = 4 per group): control (vehicle), TAZ (100 mg/kg, oral gavage), DOX (10 mg/kg, intraperitoneal injection), TAZ + DOX (a combination of the two), and Lip (equivalent dose, intravenous). Randomization was performed after tumor establishment to ensure comparable baseline tumor sizes across groups. The treatment protocol consisted of an initial 1-week phase with TAZ or TAZ-Lip administered three times, followed by the co-administration of DOX or DOX-Lip on specified days. Tumor volumes and body weights were measured every 2 days, and the tumor volume was calculated as V = (length × width^2^)/2. At the study endpoint, the mice were sacrificed, tumors were excised for further analyses, and routine blood parameters were determined using an automated biochemical analyzer (Genrui).

For toxicity assessment, major organs (heart, liver, spleen, lung, and kidney) were harvested, fixed, and subjected to histological analysis. Tissue sections were processed and stained with hematoxylin and eosin (H&E) according to standard protocols. In addition, cardiac tissue was further evaluated using Masson’s trichrome staining to assess myocardial fibrosis.

### 2.18 Immunohistochemistry

Paraffin-embedded tissue sections were deparaffinised, rehydrated, and subjected to antigen retrieval. After permeabilization and blocking, sections were probed with antibodies against Ki67, EZH2, and γH2AX overnight at 4 °C. Signals were detected using a biotinylated secondary antibody and DAB chromogen followed by digital slide scanning.

### 2.19 TUNEL staining

Apoptotic cells in tumor sections were detected using a commercial TUNEL kit according to the supplied protocol and imaged by GLSM.

### 2.20 Statistical analysis

All assays were conducted with at least three independent biological replicates. Data are presented as mean ± SD and were analyzed using GraphPad Prism (v10). Comparisons between two groups were conducted by unpaired two-tailed Student’s t-tests, while multiple groups were analyzed by one-way or two-way ANOVA. Quantitative data from fluorescence imaging and immunoblotting were analyzed using ImageJ and normalized to controls. Statistical significance was defined as P < 0.05, with exact p-values indicated in the figure legends.

## 3 Results

### 3.1 High EZH2 expression is associated with poor prognosis in BRCA

**FIGURE 1:**
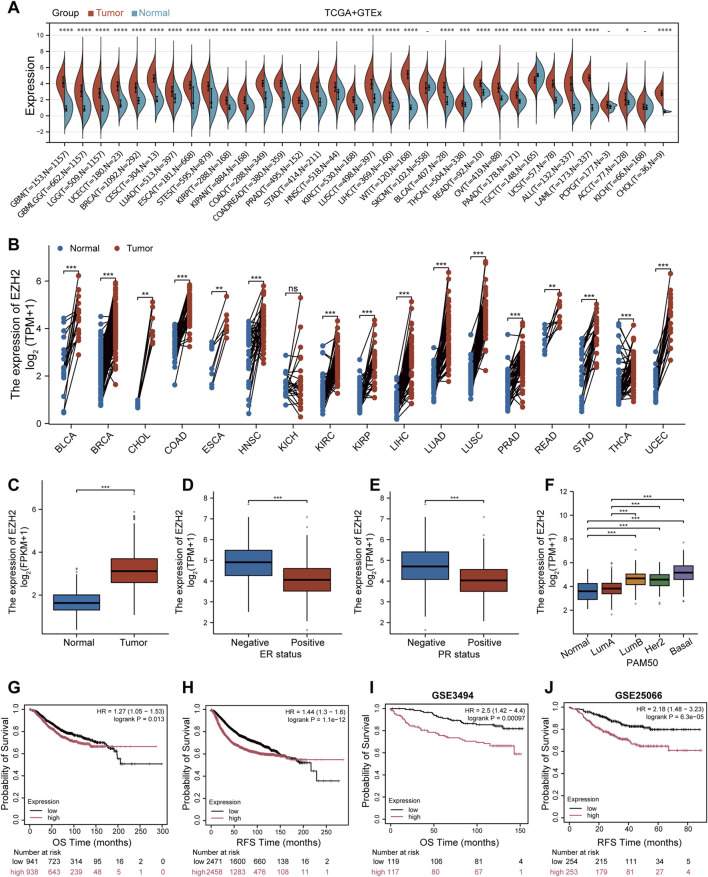
EZH2 is overexpressed in BRCA and predicts poor clinical outcome **(A)** Pan-cancer profiling of EZH2 across malignancies using TCGA and GTEx datasets. **(B)** Paired comparison of EZH2 levels in tumors versus matched adjacent normal tissues. **(C)** EZH2 expression in normal versus tumor breast tissues. **(D–F)** EZH2 expression in BRCA stratified by estrogen receptor (ER) status **(D)**, progesterone receptor (PR) status **(E)** and PAM50 subtype **(F)**. **(G–J)** Kaplan–Meier survival curves comparing EZH2-high and EZH2-low groups: overall survival (OS; **(G,I)** and recurrence-free survival (RFS; **(H,J)** derived from the Kaplan–Meier Plotter database. *P < 0.05, **P < 0.01, ***P < 0.001, ****P < 0.0001; ns, not significant.

To investigate the clinical significance of EZH2 in BRCA, we first profiled its transcript levels across human cancers using TCGA and GTEx datasets. Among diverse malignancies, EZH2 was markedly overexpressed, with one of the most pronounced elevations observed in BRCA (Figure 1A; Supplementary Figure S1). Paired analysis further confirmed the significant upregulation in tumors compared to matched normal tissues (Figure 1B). Consistently, EZH2 expression was robustly increased in BRCA samples from TCGA cohort relative to that in normal controls (Figure 1C). When stratified by hormone receptor status, estrogen receptor (ER)-negative and progesterone receptor (PR)-negative tumors exhibited higher EZH2 levels than did receptor-positive tumors (Figures 1D,E). Patients aged ≤60 years showed increased EZH2 levels compared to those in older patients (Supplementary Figure S1B). However, the HER2 status did not significantly affect EZH2 expression (Supplementary Figure S1C). Higher EZH2 expression was also observed in tumors with pathological grade II relative to grade I, and in T2-stage tumors compared to T1-stage tumors (Supplementary Figure S1D,E). Notably, PAM50 intrinsic subtyping identified the highest EZH2 expression in basal-like tumors, a surrogate for triple-negative breast cancer, relative to luminal A, luminal B, and HER2-enriched subtypes (Figure 1F). Collectively, these findings suggest a strong association between EZH2 overexpression and aggressive tumor phenotypes.

Kaplan-Meier analysis was used to evaluate the prognostic value of EZH2 in BRCA. High EZH2 expression was significantly associated with unfavorable clinical outcomes, including shorter overall survival (HR = 1.27, 95% CI: 1.05–1.53, *P* = 0.013; Figure 1G), reduced recurrence-free survival (HR = 1.44, 95% CI: 1.3–1.6, *P* < 0.0001; Figure 1H), and diminished post-progression survival (HR = 1.47, 95%CI: 1.17–1.86, *P* = 0.0011, Supplementary Figure S1F), and decreased distant metastasis-free survival (HR = 1.37, 95%CI: 1.17–1.6, *P* < 0.0001, Supplementary Figure S1G). These prognostic associations were independently validated in external datasets from GEO (GSE3494, GSE25066, GSE2034) and EBI ArrayExpress (E-MTAB-365), confirming its robustness as a biomarker of poor outcome (Figures 1I,J; Supplementary Figure S1H,I). Together, these data identify EZH2 overexpression as a molecular hallmark of BRCA with potential prognostic and therapeutic relevance.

### 3.2 DOX induced EZH2 expression in BRCA

**FIGURE 2:**
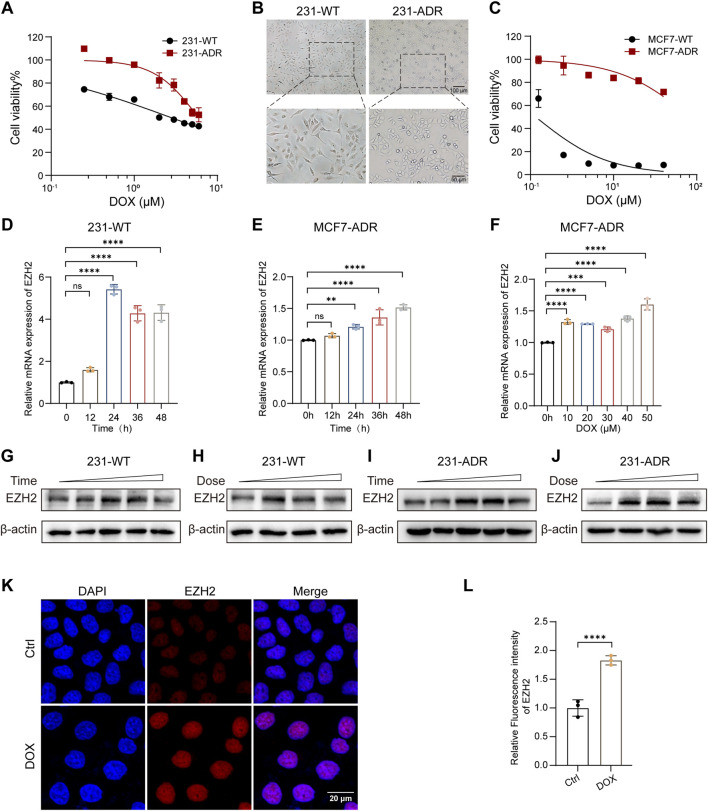
DOX induces EZH2 upregulation in BRCA **(A)** Cell viability of 231-WT (parental) and 231-ADR (DOX-resistant) cells after DOX treatment. **(B)** Bright-field images of 231-WT (left) and 231-ADR (right). Scale bars: 100 μm (top); 50 μm (bottom). **(C)** Cell viability of MCF7-WT and MCF7-ADR cells following DOX treatment. **(D)** EZH2 mRNA levels in 231-WT cells exposed to DOX over time (n = 3). **(E,F)** EZH2 mRNA levels in MCF7-ADR cells following DOX treatment: time course **(E)** and dose response **(F)** (n = 3). **(G–J)** EZH2 protein levels in 231-WT **(G,H)** and 231-ADR cells **(I,J)** following DOX exposure: time course **(G,I)** and dose-response **(H,J)**. (**K,L**) Representative GLSM images **(K)** and corresponding quantification (**L**) of EZH2 in 231-ADR cells (n = 3). Scale bar: 20 μm ****P < 0.0001.

To explore the potential involvement of EZH2 in chemoresistance, we established a DOX-resistant BRCA cell line MDA-MB-231-ADR (231-ADR) for subsequent mechanistic studies. Acquisition of resistance was confirmed by CCK-8 assays, showing markedly increased IC_50_ values in resistant versus parental cells (4.48 μM vs. 0.61 μM for 231-ADR and 231-WT (parental), respectively; Figure 2A). Morphologically, resistant cells displayed reproducible morphological changes, adopting a rounded cluster-forming phenotype distinct from the elongated morphology of parental cells (Figure 2B). In addition, a second DOX-resistant cell line, MCF7-ADR, was included and similarly displayed increased resistance to DOX (Figure 2C).

Next, we examined whether DOX exposure alters EZH2 expression. qRT–PCR analysis revealed a time-dependent increase in EZH2 mRNA levels in DOX-sensitive cells following treatment (Figure 2D). Interestingly, dose–response experiments revealed a biphasic pattern in which low DOX concentrations upregulated EZH2, whereas higher doses produced a progressive decline (Supplementary Figure S2A–D). A similar trend was observed for resistant cells (Supplementary Figure S2E–H). Notably, EZH2 expression remained persistently elevated for up to 48 h post-treatment in 231-ADR cells (Supplementary Figure S2I–L), indicating a sustained transcriptional activation during prolonged drug exposure. Consistently, in the MCF7-ADR model, DOX also induced EZH2 expression in both a time- and dose-dependent manner (Figures 2E,F). Western blotting further confirmed that DOX treatment increased EZH2 protein levels in both sensitive and resistant lines in a time- and dose-dependent manner (Figures 2G–J). Immunofluorescence analysis comparing control and DOX-treated cells showed enhanced EZH2 signal following treatment (Figures 2K,L). Together, these data indicate that DOX elicits adaptive upregulation of EZH2 across multiple BRCA models, including resistant cells, suggesting a potential role in adaptive chemoresistance.

### 3.3 Pharmacological inhibition of EZH2 overcomes DOX resistance in BRCA

**FIGURE 3:**
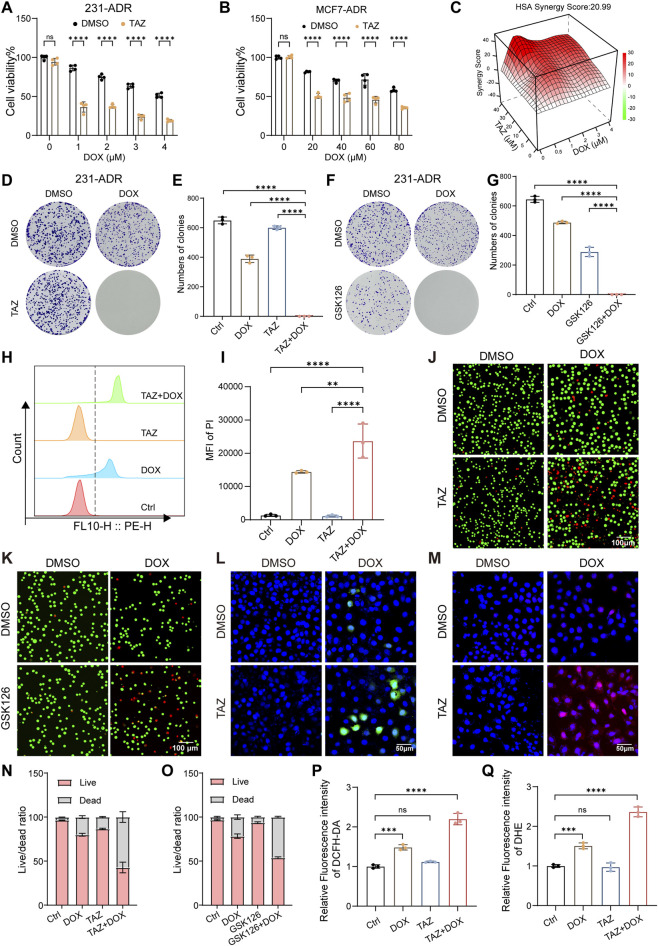
Targeting EZH2 reverses chemotherapy resistance in BRCA **(A,B)** Dose response of DOX-induced cytotoxicity in 231-ADR **(A)** and MCF7-ADR **(B)** cells (n = 4). **(C)** Synergy plot using an HSA model for 231-ADR cells treated with TAZ and DOX. **(D–G)** Representative images **(D,F)** and quantitative analysis **(E,G)** of colony formation assays in 231-ADR cells (n = 3). **(H,I)** PI staining in 231-ADR cells after 24 h of indicated treatment assessed by flow cytometry **(H)** with quantification analysis **(I)** (n = 3). (**J,K,N,O**) Live/dead staining of 231-ADR cells pretreated with TAZ or GSK126 followed by DOX; representative images **(J,K)** and quantification (**N,O**). Scale bar: 100 µm. (**L,P**) Representative images (**L**) and quantification (**P**) of intracellular ROS levels detected by DCFH-DA fluorescence in 231-ADR cells (n = 3). Scale bar: 50 μm. (**M,Q**) Representative images (**M**) and quantification (**Q**) of superoxide anion levels measured by dihydroethidium (DHE) fluorescence in 231-ADR cells (n = 3). Scale bar: 10 μm **P < 0.01, ***P < 0.001, ****P < 0.0001; ns, not significant.

Having established that DOX induces EZH2 expression, we next investigated whether targeting EZH2 can reverse chemoresistance. BRCA cells were pretreated with TAZ for 3 days, followed by DOX exposure. In DOX-resistant cells, TAZ pretreatment markedly enhanced DOX-induced cytotoxicity compared with either agent alone, as demonstrated in both 231-ADR and MCF7-ADR models (Figures 3A,B). Consistently, comparable sensitization effects were observed using another EZH2i, GSK126, in both resistant cell lines (Supplementary Figure S3A,B), confirming that chemo-sensitization is not limited to a single inhibitor. Interestingly, TAZ also sensitized the parental DOX-sensitive cell lines, albeit to a lesser extent (Supplementary Figure S3C,D), suggesting a broader role of EZH2 in modulating the DOX response. Drug interaction analyses further demonstrated strong synergism between TAZ and DOX, with the highest single-agent (HSA) scores ≥20 in 231-ADR cells (Figure 3C). This synergism was further corroborated by multiple computational models including Loewe, Bliss, and ZIP (Supplementary Figure S3E–G). Similar effects were observed when TAZ was replaced with GSK126, thus underscoring the reproducibility and EZH2 specificity of this effect (Supplementary Figure S3H–K). In addition, full drug response matrices were generated for 231-ADR cells treated with TAZ or GSK126 (Supplementary Figure S3M,N). Consistent findings were observed in MCF7-ADR cells, where synergistic effects were further supported by HSA analysis (Supplementary Figure S3L) and corresponding dose–response matrices (Supplementary Figure S3O).

Clonogenic assays demonstrated that while individual agents had modest effects, the TAZ-DOX combination almost completely abolished colony formation, indicating sustained suppression of proliferative capacity (Figures 3D–G). To quantify cell death, we performed propidium iodide (PI) staining by flow cytometry in 231-ADR cells. The combination treatment significantly increased the proportion of PI-positive cells compared with either agent alone (Figures 3H,I). Live/dead cell imaging using AM/PI staining further supported these findings, visually demonstrating extensive cell death only upon cotreatment of EZH2i and DOX (Figures 3J,K,N,O). Given that DOX promoted ROS accumulation, we examined whether EZH2 inhibition augments this process (Kciuk et al., 2023). DCFH-DA fluorescence assays revealed that TAZ pre-treatment significantly augmented DOX-induced ROS production (Figures 3L,P). Dihydroethidium staining consistently demonstrated marked superoxide anion accumulation in the combination regimen (Figures 3M,Q). Collectively, these findings demonstrate that the pharmacological inhibition of EZH2 restores DOX sensitivity and enhances cytotoxicity in BRCA cells, accompanied by increased oxidative stress.

### 3.4 EZH2 blockade enhances DOX-Induced DNA damage and cytotoxicity

**FIGURE 4:**
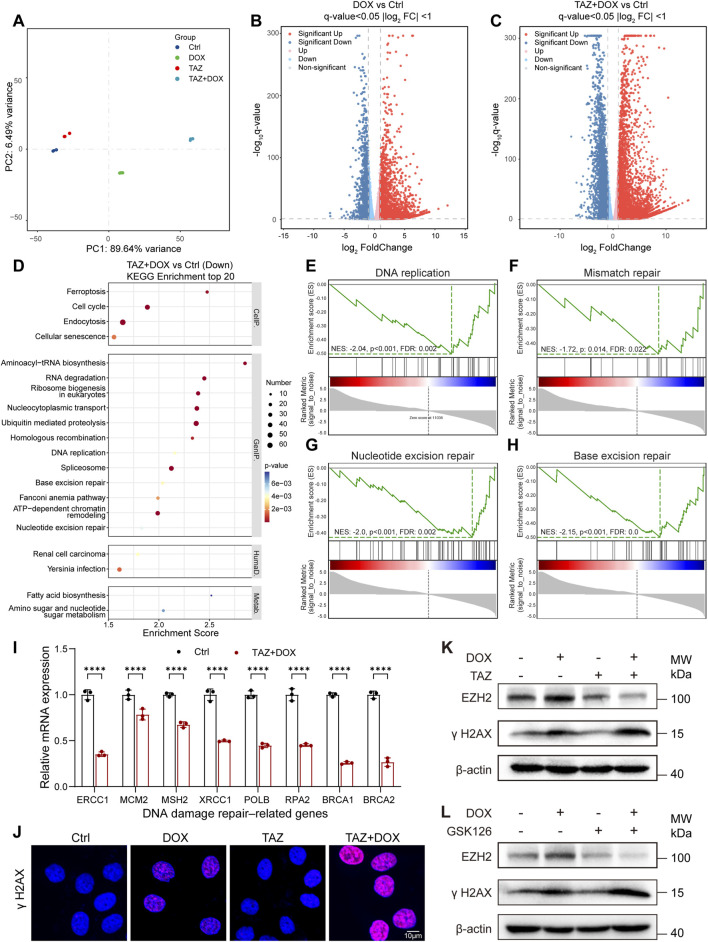
Targeting EZH2 promotes DOX-induced cell death by downregulating DNA damage repair pathways **(A)** Principal component analysis (PCA) of 231-ADR cells under the indicated treatments. **(B,C)** Volcano plots of DEGs in 231-ADR cells treated with DOX **(B)** or TAZ + DOX **(C)** versus control. **(D)** KEGG pathway enrichment analysis of downregulated DEGs. **(E–H)** GSEA charts for KEGG categories DNA replication **(E)**, mismatch repair **(F)**, nucleotide excision repair **(G)**, and base excision repair pathway **(H)**. **(I)** qRT–PCR validation of representative DNA repair–related genes in 231-ADR cells (n = 3). **(J)** Representative immunofluorescence images of γH2AX in 231-ADR cells (n = 3). Scale bar: 10 µm. (**K,L**) Immunoblot for γH2AX and EZH2 in 231-ADR cells, with β-actin as a loading control (n = 3).

To further elucidate the mechanism by which EZH2 blockade alleviates DOX resistance, we performed whole-transcriptome RNA sequencing (RNA-seq) analysis on 231-ADR cells treated with TAZ + DOX, DOX, and TAZ versus controls. Principal component analysis (PCA) revealed significant transcriptomic differences across the four groups (Figure 4A). Differential expression analysis revealed 4,634 significantly dysregulated genes (1,355 downregulated and 3,279 upregulated; false discovery rate (FDR) < 0.05) in the DOX-treated group compared to the controls, and 9,262 significantly dysregulated genes (3,339 downregulated and 5,923 upregulated; FDR <0.05) in the TAZ-DOX combination group compared to the controls (Figures 4B,C). KEGG enrichment analysis identified significant suppression of pathways related to DNA damage repair in the TAZ + DOX treatment group, including DNA replication, base excision repair, nucleotide excision repair, and ATP-dependent chromatin remodeling pathways (Figure 4D). Consistently, GSEA revealed that pathways involved in DNA damage, such as DNA replication, mismatch repair, nucleotide excision repair, and base excision repair pathways, were significantly suppressed, indicating a downregulation of DNA damage repair–related transcriptional programs in 231-ADR cells after TAZ + DOX combination therapy (Figures 4E–H; Supplementary Table S3).

Based on these findings, we hypothesized that EZH2 inhibition exacerbated DOX-induced DNA damage. To validate these transcriptomic results, qRT–PCR analysis of representative DNA repair–related genes was performed in TAZ + DOX versus control groups. Consistent with RNA-seq data, key DNA repair genes were significantly downregulated following combination treatment (Figure 4I). To determine whether these transcriptional changes translate into functional DNA damage, γH2AX levels were assessed by immunofluorescence. DOX alone induced modest DNA damage, as indicated by weak γH2AX staining, whereas co-treatment with TAZ markedly increased γH2AX signal (Figure 4J; Supplementary Figure S4C). This effect was consistently observed across models and conditions. In 231-ADR cells, GSK126 in combination with DOX similarly increased γH2AX fluorescence compared with DOX alone (Supplementary Figure S4A,D). In addition, in the luminal-type MCF7-ADR model, TAZ combined with DOX also enhanced γH2AX accumulation (Supplementary Figure S4B,E). Western blotting analysis further confirmed increased γH2AX levels and reduced EZH2 expression in the combination therapy group (Figure 4K; Supplementary Figure S4F–G). Similar results were observed with GSK126 (Figure 4L; Supplementary Figure S4H–I). Thus, EZH2 inhibition enhanced DOX-induced cytotoxicity, accompanied by transcriptional suppression of DNA repair–associated pathways and increased DNA damage.

### 3.5 TAZ induced metabolic vulnerability of BRCA cells

**FIGURE 5:**
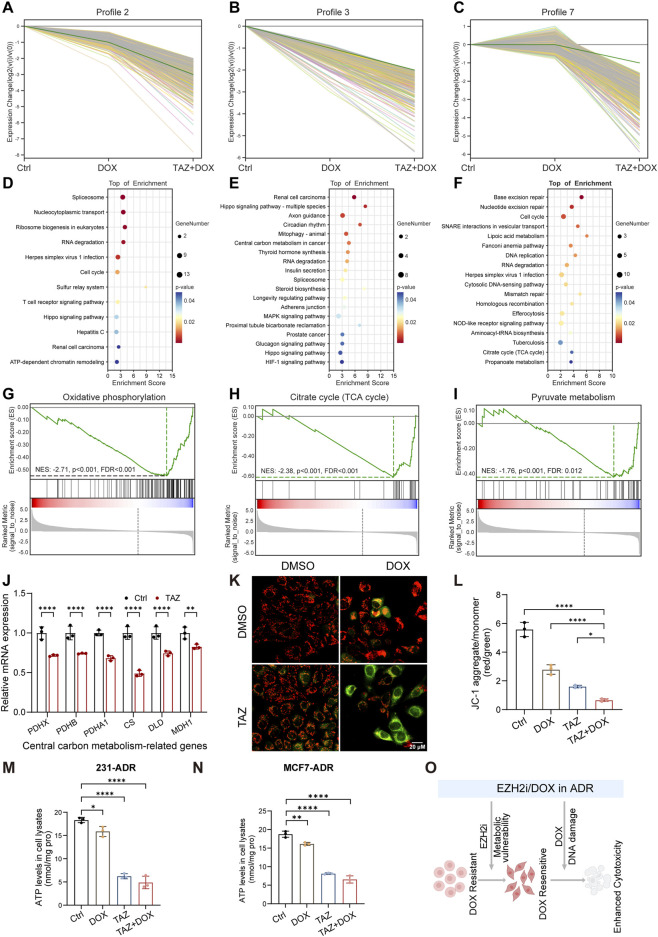
TAZ induced metabolic vulnerability of BRCA cells. (**A-C**) Time-series analysis across selected gene profiles under the indicated treatments. (**D-F**) KEGG pathway enrichment analysis of DEGs corresponding to the profiles in (**A–C**). (**G-I**) GSEA charts for KEGG categories oxidative phosphorylation (**G**), tricarboxylic acid (TCA) cycle (**H**), and pyruvate metabolism (**I**) in 231-ADR cells treated with TAZ versus control. (**J**) qRT–PCR validation of representative genes involved in central carbon metabolism in 231-ADR cells (n = 3). (**K,L**) Representative JC-1 staining images (**K**) and corresponding quantification (**L**) of mitochondrial membrane potential in 231-ADR cells (n = 3). Scale bar: 20 µm. (**M,N**) Intracellular ATP levels in 231-ADR (**M**) and MCF7-ADR (**N**) cells under the indicated treatments (n = 3). (**O**) Schematic illustration of EZH2 mediated metabolic vulnerability and its role in enhancing DNA damage. *P < 0.05, **P < 0.01, ****P < 0.0001.

To explore how EZH2 inhibition sensitizes cells to DOX, we analyzed transcriptional changes associated with EZH2 in 231-ADR cells. Genes downregulated by DOX (vs. control) were intersected with those upregulated upon TAZ treatment in the presence of DOX, yielding 190 overlapping genes enriched in several functional pathways (Supplementary Figure S5A,B). However, these results did not fully capture the transcriptional dynamics of the combination treatment. We next performed time-series analysis, which revealed that TAZ potentiated DOX-induced transcriptional changes in a subset of genes (Figures 5A–C), which were mainly associated with DNA damage repair and central carbon metabolism (Figures 5D–F). Gene Ontology (GO) enrichment analysis highlighted decreased DNA repair, DNA binding, chromosome segregation, RNA binding, and protein binding in the combination group compared to DOX monotherapy (Supplementary Figure S5C). KEGG and GSEA analyses showed significant suppression of DNA damage repair–related pathways, including base excision repair, mismatch repair, and nucleotide excision repair (Supplementary Figure S5D–G; Supplementary Table S3).

To further delineate the metabolic role of EZH2 inhibition, RNA-seq analysis of TAZ-treated ADR cells revealed suppression of the oxidative phosphorylation (OXPHOS), citrate cycle (TCA cycle), and pyruvate metabolism pathways (Figures 5G–I; Supplementary Table S3). qRT–PCR analysis confirmed downregulation of representative genes involved in central carbon metabolism in 231-ADR cells (Figure 5J). We hypothesized that TAZ mediates metabolic vulnerability of ADR cells, thereby rendering them more sensitive to DOX. Functionally, TAZ treatment reduced mitochondrial membrane potential, which was exacerbated upon DOX co-treatment (Figures 5K,L). Consistently, TAZ treatment significantly reduced cellular ATP levels (Figure 5M), similar effects were observed in MCF7-ADR cells, indicating that the metabolic impact of EZH2 inhibition is reproducible across models (Figure 5N).

To further characterize redox regulation, we first examined the transcriptional changes of antioxidant-related genes. qRT–PCR analysis revealed a heterogeneous expression pattern across groups. While several antioxidant genes were upregulated in response to DOX and/or TAZ treatment, their regulation was not uniform under combination treatment, with some genes remaining elevated and others showing reduced expression (Supplementary Figure S6A–E). Given this heterogeneous profile, we next assessed antioxidant capacity at the functional level. Superoxide dismutase (SOD) activity was decreased in DOX-treated cells but increased following TAZ treatment, indicating a compensatory antioxidant response (Supplementary Figure S6F). In the combination group, SOD activity remained elevated relative to controls but was lower than that in the EZH2i group (Supplementary Figures S6F–H).

In summary, EZH2 inhibition disrupted mitochondrial energy homeostasis and created a metabolic vulnerability that potentially rendered cells more susceptible to DOX-induced cytotoxicity (Figure 5O).

### 3.6 Fabrication of pH- responsive nanoliposomes for DOX/TAZ delivery

Given the robust synergistic cytotoxicity observed with TAZ and DOX *in vitro*, we evaluated their combined antitumor efficacy *in vivo*. However, the clinical utility of DOX is often limited by its dose-dependent systemic toxicity (Linders et al., 2024; Marzochi et al., 2023). Previous studies have reported that liposome (Lip) delivery platforms have emerged as a promising approach for improving tumor targeting efficiency (Chen J. et al., 2024). Thus, we developed a tumor-acidic microenvironment-responsive liposomal delivery system (DOX/TAZ-Lip) incorporating cholesteryl hemisuccinate as a pH-sensitive component to enhance tumor-specific drug release.

**FIGURE 6:**
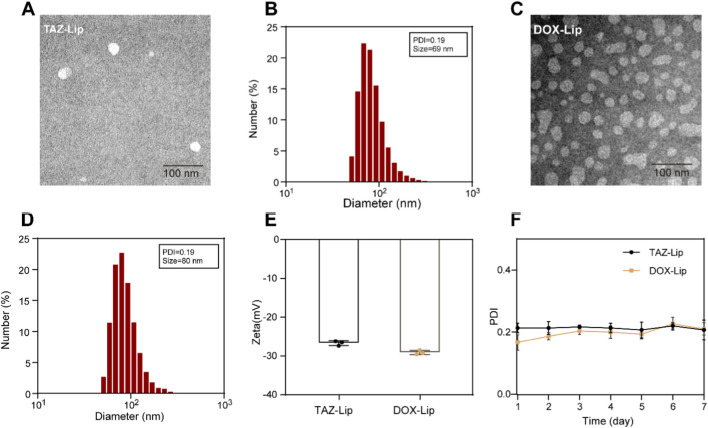
Characterization of TAZ/DOX-Lip nanoparticles. **(A,C)** TEM images of TAZ-Lip **(A)** and DOX-Lip **(C)**, Scale bar: 100 nm. **(B,D)** Size distribution of TAZ-Lip **(B)** and DOX-Lip **(D)**. **(E)** Zeta potentials of TAZ-Lip and DOX-Lip (n = 3). **(F)** PDI of TAZ-Lip and DOX-Lip NPs measured over 7 days (n = 3).

Transmission electron microscopy (TEM) confirmed that both DOX-Lip and TAZ-Lip exhibited spherical morphologies with uniform size and good dispersity (Figures 6A,C). Dynamic light scattering (DLS) measurements showed hydrodynamic diameters of approximately 69 nm for TAZ-Lip and 80 nm for DOX-Lip, with Zeta potentials of −26.5 mV and −29.6 mV, respectively (Figures 6B,D,E). Lip maintained excellent stability over 7 days, as evidenced by negligible changes in the polydispersity index (Figure 6F). The drug encapsulation efficiency, determined by UV-Vis spectroscopy (480 nm) for DOX and high-performance liquid chromatography (HPLC) for TAZ, reached 72% and 80%, respectively.

Collectively, these data confirmed the successful preparation of a stable DOX/TAZ delivery system with a high drug-loading capacity suitable for *in vivo* administration.

### 3.7 Antitumor efficacy *in vivo*

**FIGURE 7:**
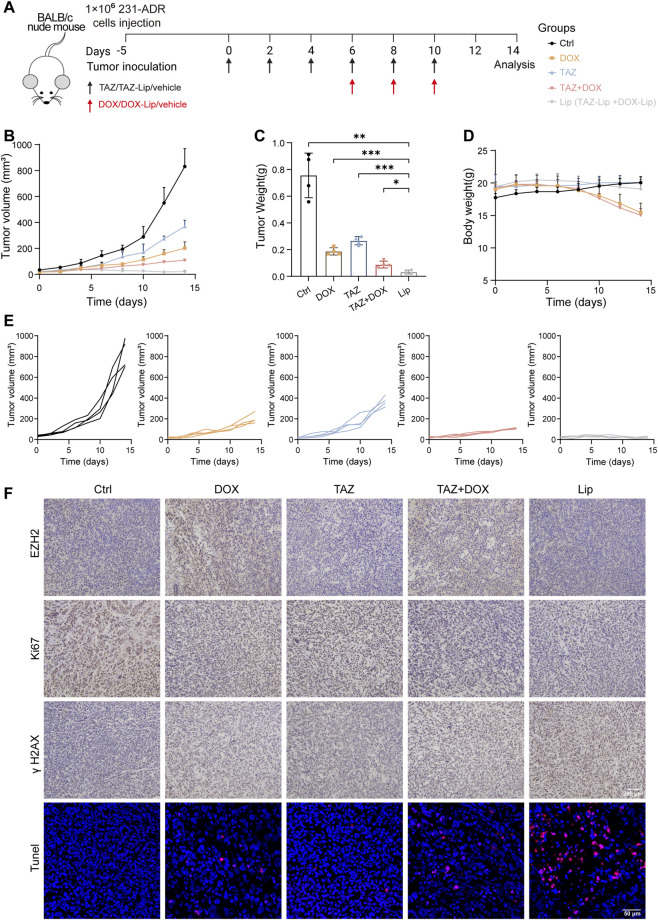
*In vivo* antitumor efficiency assessments **(A)** Diagram of the *in vivo* therapeutic schedule. **(B,C)** Tumor growth curve **(B)** and terminal tumor weights **(C)** for each treatment group (n = 4 mice per group). **(D)** Body weight changes of mice during treatment (n = 4 per group). **(E)** Tumor growth curves of 231-ADR mice model under the indicated treatments. **(F)** IHC analysis of EZH2, Ki67 and γH2AX expression and TUNEL immunofluorescence staining in tumor tissues after different treatments. Scale bar: 200 μm (top). Scale bar: 50 μm (bottom). *P < 0.05, **P < 0.01, ***P < 0.001.

Next, we evaluated the antitumor efficacy of the TAZ–DOX combination strategy in a 231-ADR xenograft model. The mice were randomized into five treatment groups: control (Ctrl), TAZ alone, DOX alone, TAZ + DOX (free drug combination), and Lip (TAZ-Lip and DOX-Lip) (Figure 7A). Tumor growth was monitored throughout the study and was quantitatively assessed at the endpoint. Notably, while TAZ monotherapy showed limited efficacy and DOX alone elicited only modest tumor suppression in this resistant setting, the TAZ + DOX combination significantly impeded tumor progression (Figures 7B,C,E; Supplementary Figure S7A). Interestingly, the Lip group exhibited the most potent antitumor activity, nearly ablating tumor growth and reducing the final tumor volume to 15.7% of the control (Figures 7B,C). However, mice treated with free DOX, either alone or in combination with TAZ, displayed substantial weight loss starting at day 8, culminating in a final body weight loss of 23% and 25% of the control group, respectively (Figure 7D). These groups also showed evidence of hematological toxicity (Supplementary Figure S7B–H). H&E staining revealed no obvious histopathological abnormalities in major organs (Supplementary Figure S8A). In contrast, Masson staining showed collagen fiber deposition in cardiac tissues of DOX-treated mice, which was also observed in the TAZ + DOX group, indicating early DOX-associated cardiotoxicity (Supplementary Figure S8B). No obvious collagen deposition was detected in the TAZ or Lip groups. Together, these results indicate that liposomal delivery improves the safety profile of DOX while maintaining antitumor efficacy.

To delineate the underlying mechanisms, we performed histopathological and molecular analyses of the resected tumor tissues (Figure 7F). Immunohistochemical staining revealed a significant reduction in Ki67-positive cells in the Lip group, indicating potent anti-proliferative effects. TUNEL staining further confirmed a substantial increase in apoptosis, which was consistent with our *in vitro* cytotoxicity data. DOX monotherapy upregulated EZH2 expression, which was reversed by TAZ co-administration, with the most profound EZH2 suppression observed in the Lip group. Furthermore, the combination strategy involving TAZ resulted in upregulated γH2AX expression compared to that in the control group, indicating robust DNA damage induction (Figure 7F). Together, the TAZ-DOX combination strategy demonstrated antitumor efficacy *in vivo*, and the liposomal delivery system could mitigate toxicity *in vivo*.

## 4 Discussion

EZH2 is widely recognized as a key epigenetic driver of tumor progression and therapy resistance; however, its specific contribution to DOX chemoresistance in BRCA remains unclear. In this study, we identified a critical role of EZH2 in sustaining the DOX-resistant phenotype in BRCA and demonstrated that pharmacological inhibition of EZH2 potently re-sensitizes resistant cells to DOX through converging effects on oxidative stress, DNA repair, and energy metabolism. Notably, this re-sensitization does not represent a reversion to a parental-like transcriptional state, but rather reflects a pharmacologically enforced stress condition in which DOX-induced cytotoxicity is amplified. These findings not only position EZH2 as an adaptive regulator of DOX resistance, but also provide a compelling rationale for combining EZH2i with conventional chemotherapy to overcome treatment failure in aggressive BRCA subtypes.

We observed that DOX treatment robustly induced EZH2 expression in both sensitive and resistant BRCA cells, which likely represents an intrinsic defense mechanism that enables tumor cells to attenuate DOX-induced oxidative and genotoxic stress. Importantly, this dynamic upregulation of EZH2 following DOX exposure underscores the need to consider the timing and sequencing when designing combination therapies. Dysregulation of EZH2 is associated with therapeutic resistance in cancer cells (Kaur et al., 2024). Previous studies have shown that EZH2 inhibition enhances the efficacy of immunotherapies and targeted agents in several tumor models (Morel et al., 2021; Isshiki et al., 2024). Our data support these observations by demonstrating that EZH2 is dynamically regulated by DOX and directly participates in the maintenance of chemoresistance in BRCA. In our models, EZH2 inhibition using TAZ or GSK126 significantly potentiated DOX-induced cytotoxicity and tumor control.

From a translational perspective, recent clinical studies have shown that TAZ exhibits a favorable safety profile but shows limited activity in solid tumors, thereby prompting increasing interest in combination strategies (Kim and Roberts, 2016; Simeone et al., 2020; von Keudell and Salles, 2021). In this context, combining EZH2 inhibition with chemotherapy represents a rational approach. However, potential toxicity interactions, particularly DOX-associated cardiotoxicity, remain important considerations (Wu et al., 2022). Liposomal formulations, by improving drug delivery and reducing systemic exposure, may offer a strategy to mitigate these limitations and enhance the therapeutic window of such combinations (Allen and Cullis, 2012).

At the mechanistic level, our findings support a model in which EZH2 maintains DOX resistance by coordinating the DNA repair capacity and metabolic fitness. EZH2 is known to regulate genes involved in cell cycle checkpoints and homologous recombination, and previous studies have shown that its inhibition impaired DNA repair in other cancer types (Liu and Yang, 2023; Wu et al., 2011; Karakashev and Zhang, 2020). Consistent with this, we observed increased γH2AX accumulation and suppression of DNA repair–related pathways when EZH2i was combined with DOX, suggesting that blocking EZH2 pushes DOX-induced DNA damage beyond the repair threshold of resistant cells.

At the same time, metabolic adaptation represents a critical component of DOX resistance. Enhanced mitochondrial function and OXPHOS activity have been reported to support ROS detoxification and survival under chemotherapy stress (He et al., 2022; Hao et al., 2025; Wu et al., 2018; Wang Y. et al., 2024). Notably, recent studies have suggested that such metabolic changes often occur alongside chromatin remodeling, with resistant cells exhibiting concurrent mitochondrial dysfunction and epigenetic alterations, sometimes coupled with autophagy-mediated survival (Xu et al., 2023; Zhou et al., 2025; Verma and Lindroth, 2025; Zhu et al., 2020). These observations point to a coordinated epigenetic–metabolic adaptation that supports the resistant phenotype.

Within this context, our findings suggest that EZH2 functions as a mechanistic link between epigenetic regulation and metabolic adaptation. Consistent with previous work showing that EZH2 can promote metabolic rewiring, including enhanced TCA cycle activity (Chen et al., 2023), we observed that EZH2 inhibition disrupted mitochondrial energy metabolism, leading to loss of membrane potential and ATP depletion. Given that DNA repair and ROS detoxification are energy-dependent processes, our data indicate that EZH2 inhibition collapses this metabolic support, thereby coupling defective DNA repair with a bioenergetic crisis (Bilkis et al., 2025; Martín et al., 2020). Therefore, rather than acting through a single pathway, EZH2 appears to support resistance by maintaining both genomic stability and metabolic balance. Notably, EZH2 also exerts non-catalytic functions that are not fully targeted by current inhibitors, which may partly explain their limited efficacy in solid tumors and highlights the need for improved therapeutic strategies (Kaur et al., 2024).

Importantly, our study does not aim to redefine EZH2 as a novel mediator of chemoresistance, but instead provides evidence that targeting EZH2 can simultaneously disrupt these interconnected processes. This dual impairment results in a combined failure of DNA repair and metabolic support, thereby creating a context in which DOX-induced damage becomes unsustainable. In this sense, the therapeutic benefit of EZH2 inhibition may lie in its ability to expose a coordinated vulnerability rather than a single downstream pathway. Together, these effects create a “double hit” on resistant cells: increased DNA damage accompanied by reduced capacity to repair or buffer.

Although our findings are encouraging, several limitations should be considered. First, the study relies on pharmacological inhibition, and potential off-target effects cannot be fully excluded despite the use of two independent EZH2 inhibitors. Second, the mechanistic link between EZH2 inhibition and coordinated disruption of DNA repair and metabolism requires further validation using genetic approaches. Third, although our *in vivo* data demonstrate promising antitumor efficacy and preliminary safety, more comprehensive studies are needed to evaluate long-term toxicity and translational potential. Finally, future studies are warranted to explore whether EZH2-related molecular signatures could serve as predictive biomarkers for patient stratification.

## 5 Conclusion

In summary, this study delineated the multifaceted role of EZH2 in driving DOX resistance through metabolic and DNA repair pathways. Pharmacological blockade of EZH2 disturbs tumor metabolism, exacerbates oxidative stress, and impairs DNA repair, collectively restoring chemosensitivity. These results not only deepen our understanding of EZH2 in chemoresistance but also open new avenues for rationally designed combination therapies.

## Data Availability

RNA-seq data are available in GEO under accession number GSE327098. All relevant data are available from the first/corresponding author on reasonable request.
