# Correction: EZH2 blockade reverses doxorubicin resistance by inducing metabolic vulnerability and enhancing DNA damage in breast cancer

**DOI:** 10.3389/fphar.2026.1882446

**Published:** 2026-06-08

**Authors:** Xiaomin Wang, Yuhang Ding, Yunxiao Mai, Ruinan Li, Xinyu Shao, Wenlong Chen, Yiming Li, Luhaoxiang Liu, Haoran Wang, Kangkang Liu, Yuanjie Niu, Jianmin Li, Guoping Xu, Yang Zhao

**Affiliations:** 1 Department of Radiology, The Second Hospital of Tianjin Medical University, Tianjin Medical University, Tianjin, China; 2 Tianjin Institute of Urology, The Second Hospital of Tianjin Medical University, Tianjin Medical University, Tianjin, China; 3 National Clinical Research Center for Geriatric Disorders, Xiangya Hospital, Central South University, Changsha, China; 4 Department of Breast Surgery, Xiangya Hospital, Central South University, Changsha, China; 5 Haihe Laboratory of Synthetic Biology, Tianjin, China

**Keywords:** breast cancer, DNA damage, doxorubicin, EZH2, metabolic vulnerability, resistance

There was a mistake in Figure 6 as published. The incorrect image was used. The corrected Figure 6 appears below.

**FIGURE 6:**
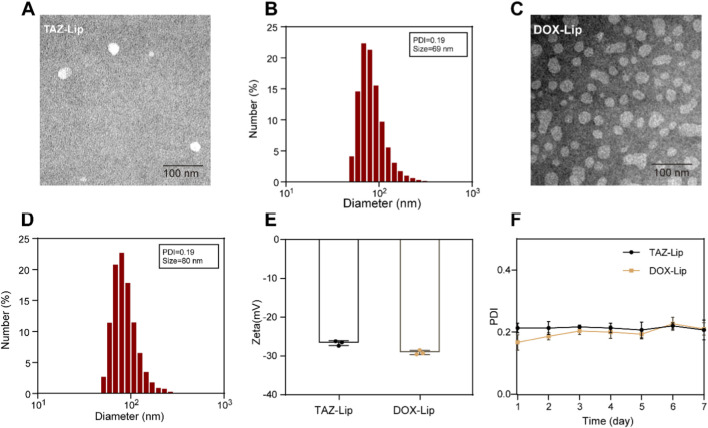
Characterization of TAZ/DOX-Lip nanoparticles. **(A,C)** TEM images of TAZ-Lip **(A)** and DOX-Lip **(C)**, Scale bar: 100 nm. **(B,D)** Size distribution of TAZ-Lip **(B)** and DOX-Lip **(D)**. **(E)** Zeta potentials of TAZ-Lip and DOX-Lip (n = 3). **(F)** PDI of TAZ-Lip and DOX-Lip NPs measured over 7 days (n = 3).

The original article has been updated.

