# Supplementary figures and images for: EZH2 blockade reverses doxorubicin resistance by inducing metabolic vulnerability and enhancing DNA damage in breast cancer
Source: Front Pharmacol. 2026 May 14;17:1786648. doi: 10.3389/fphar.2026.1786648 (PMC13216663; doi:10.3389/fphar.2026.1786648)

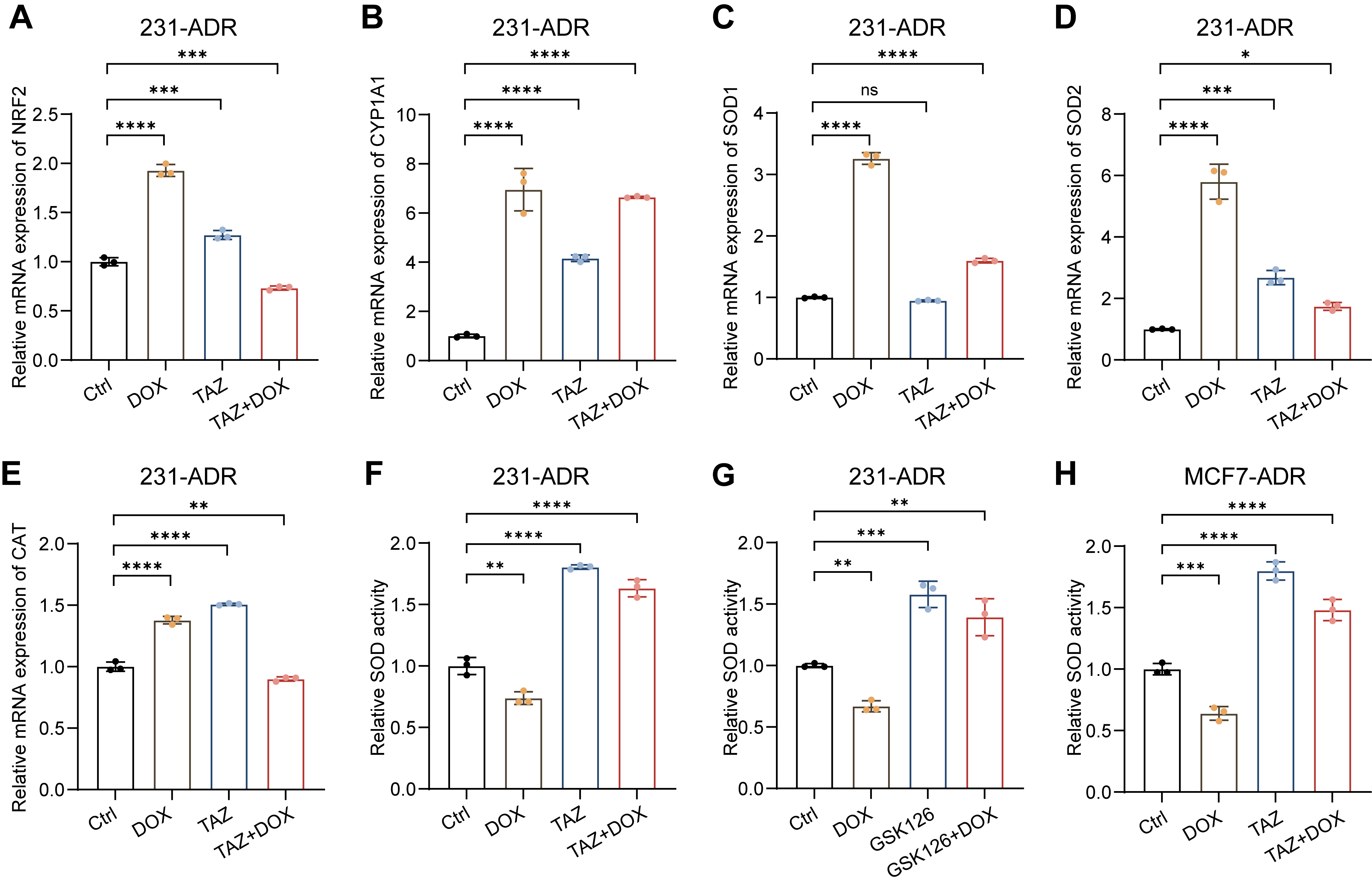

Supplement: Supplementary file 1 [file Image6.tif]

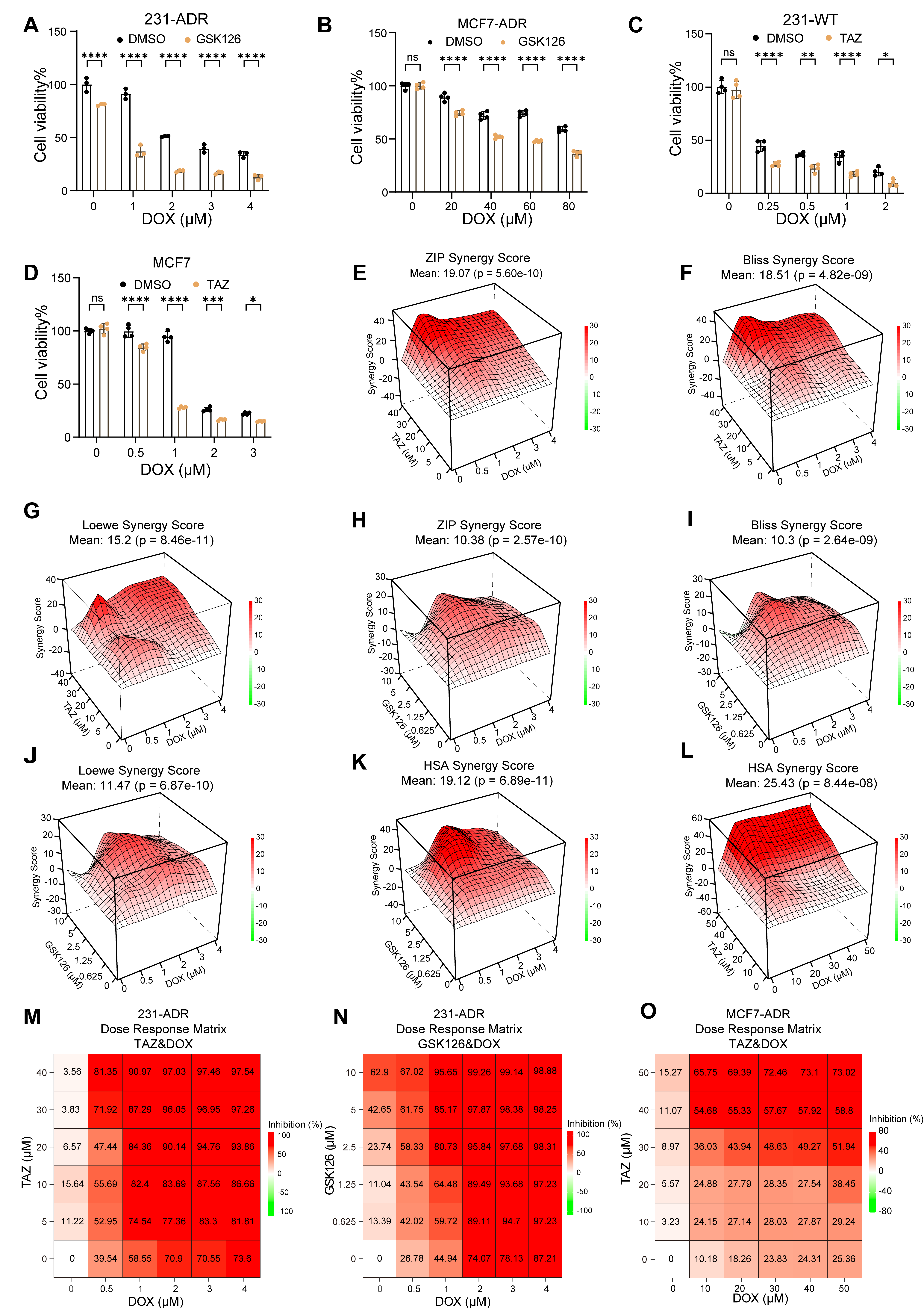

Supplement: Supplementary file 4 [file Image3.tif]

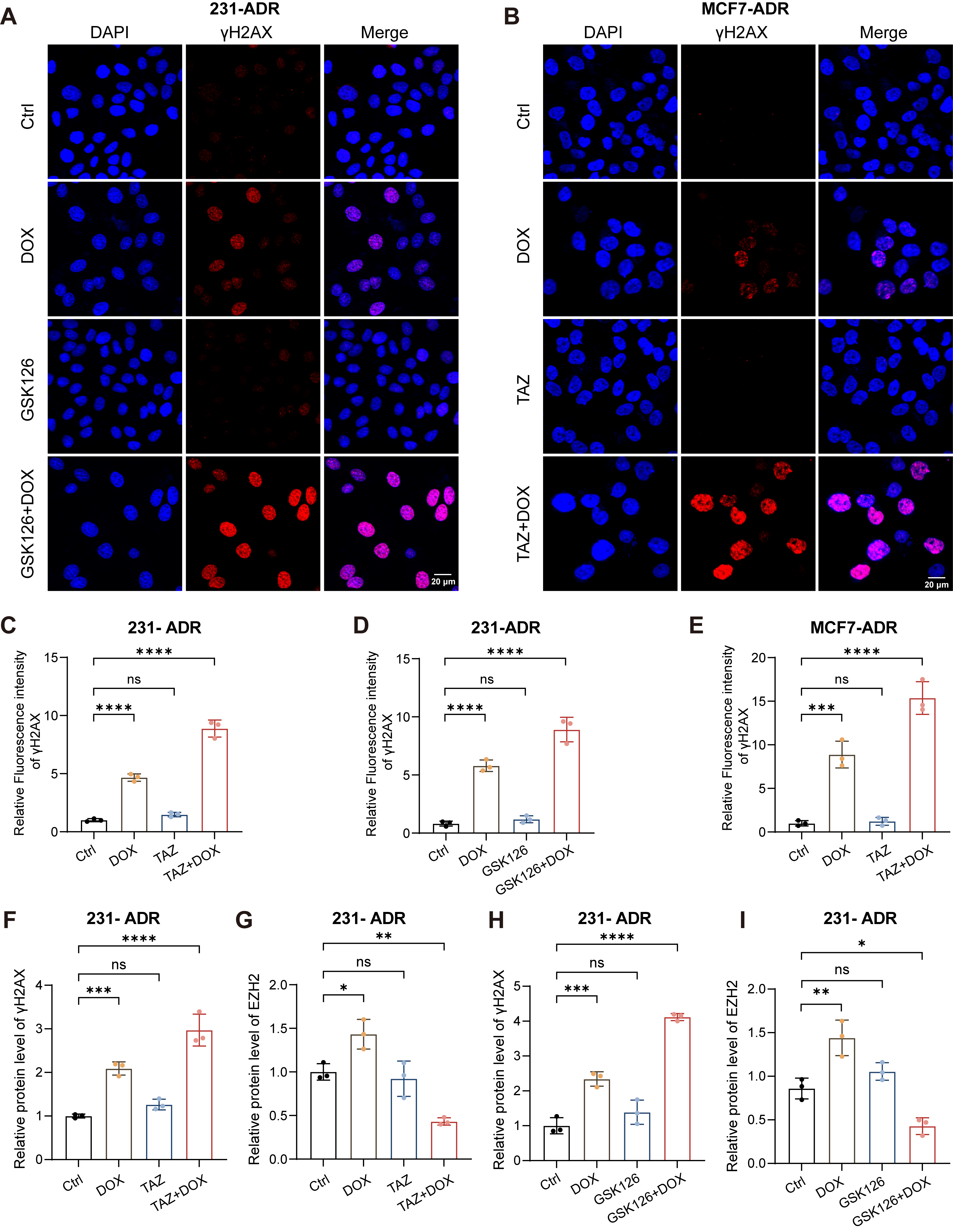

Supplement: Supplementary file 5 [file Image4.tif]

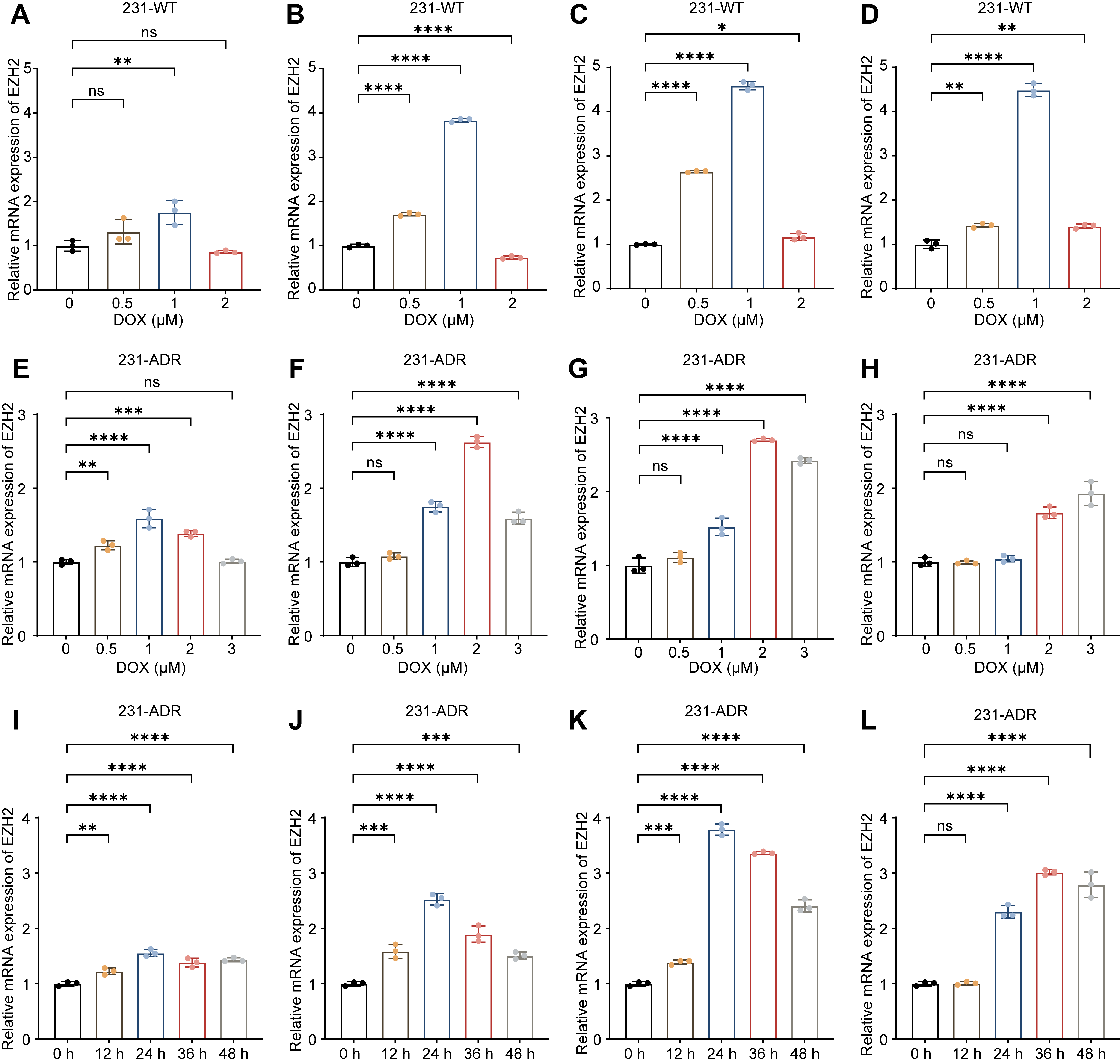

Supplement: Supplementary file 6 [file Image2.tif]

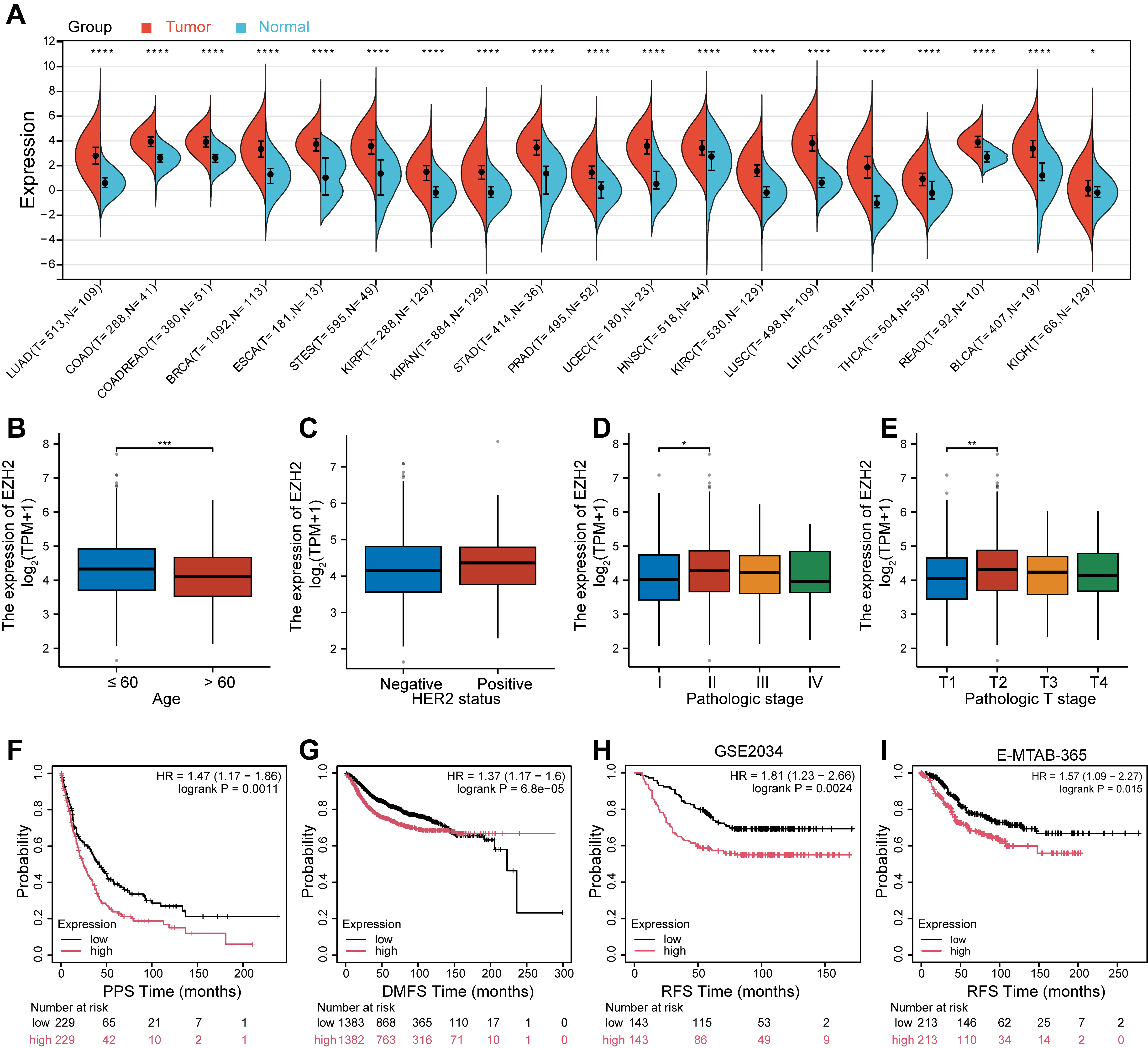

Supplement: Supplementary file 7 [file Image1.tif]

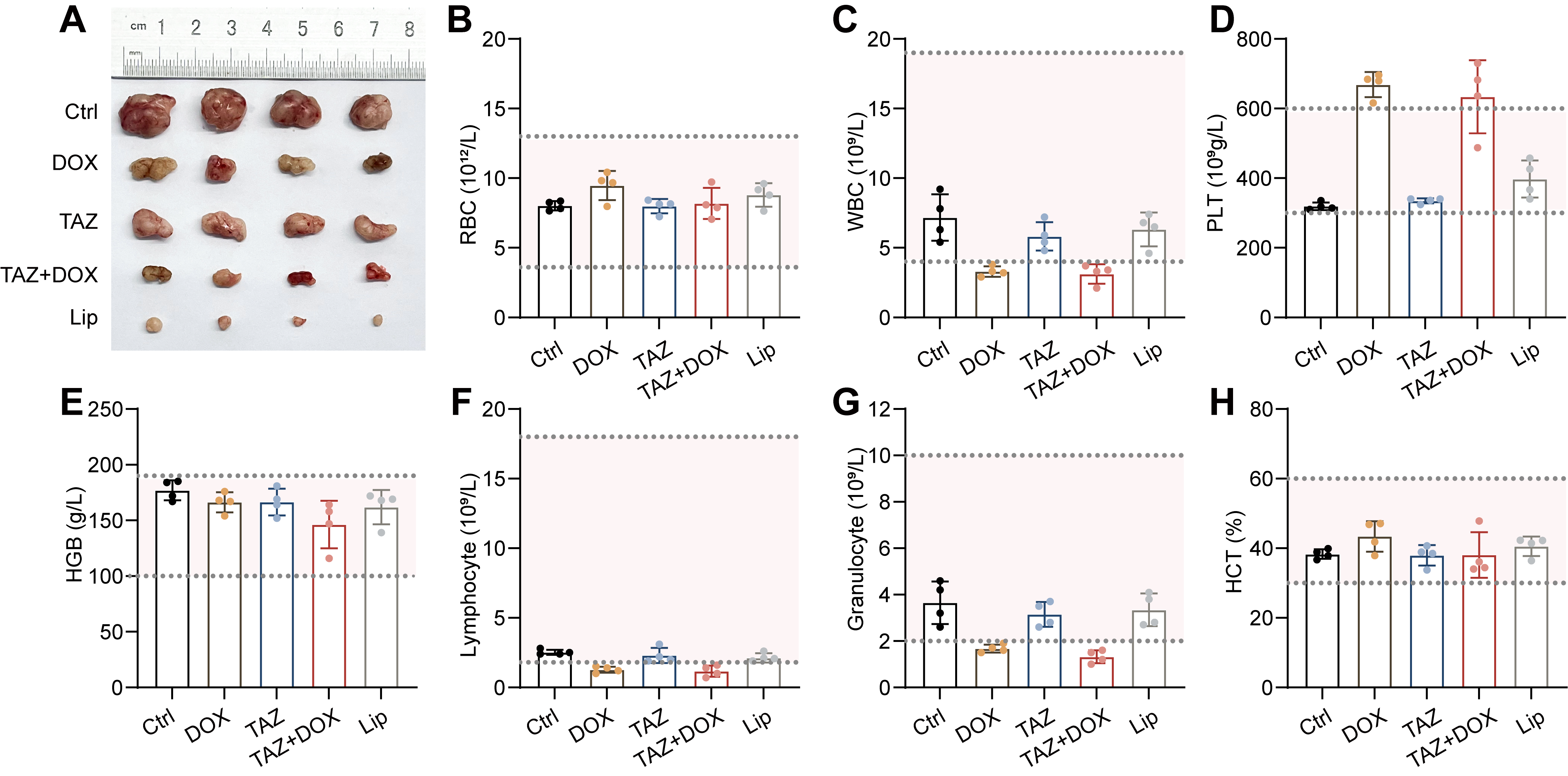

Supplement: Supplementary file 9 [file Image7.tif]

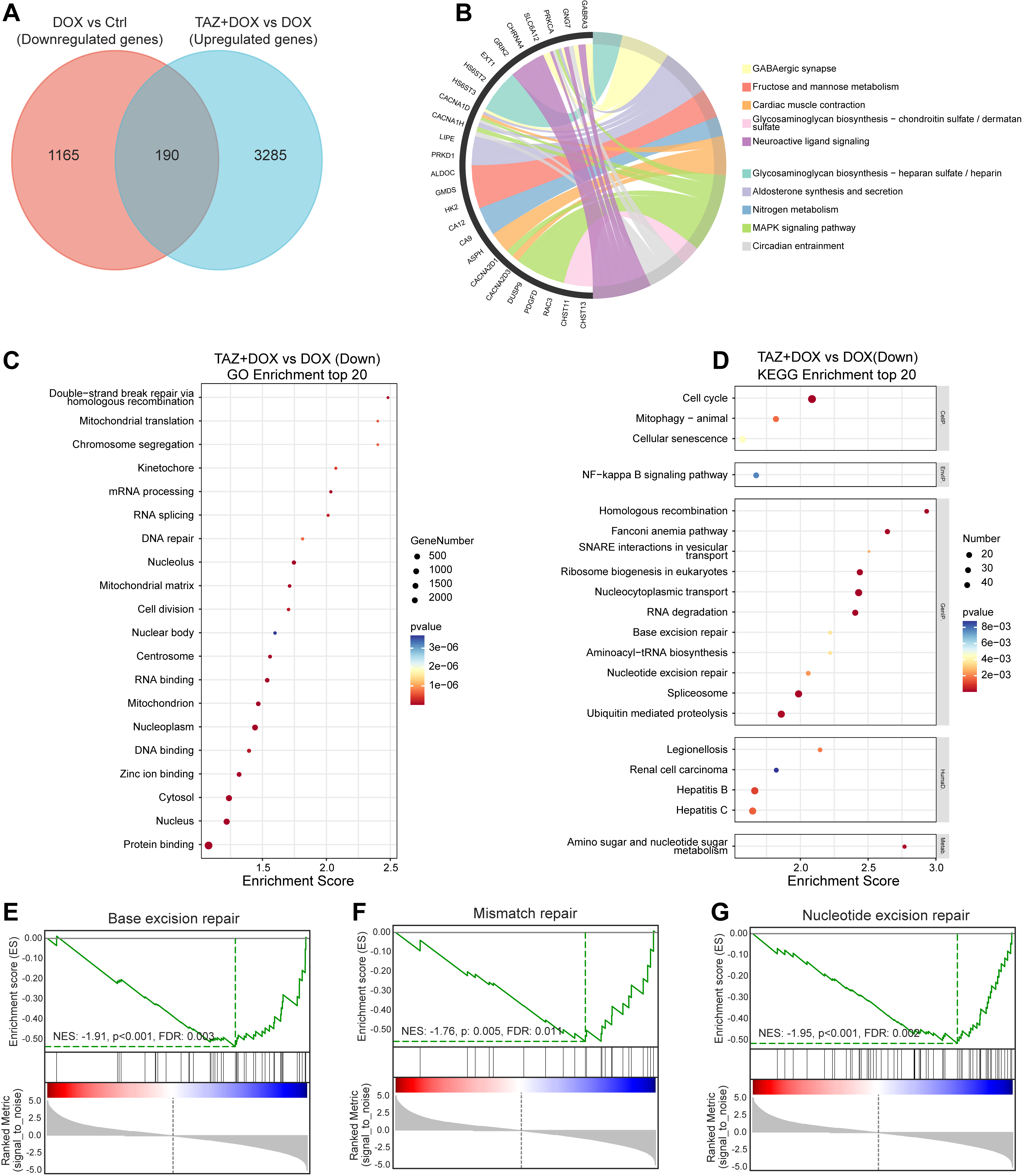

Supplement: Supplementary file 10 [file Image5.tif]
