# Supplementary material for: EZH2 blockade reverses doxorubicin resistance by inducing metabolic vulnerability and enhancing DNA damage in breast cancer
Source: Front Pharmacol. 2026 May 14;17:1786648. doi: 10.3389/fphar.2026.1786648 (PMC13216663; doi:10.3389/fphar.2026.1786648)
Supplement: Supplementary file 2 [file Supplementaryfile1.docx]

**Figure Legends of Supplementary Figures**

**Fig. S1. Pan-cancer EZH2 expression and poor prognosis in BRCA**

(**A**) Pan-cancer profiling of EZH2 across TCGA cancer types. (**B–E**) Box plots of EZH2 expression in BRCA stratified by age (**B**), HER2 status (**C**), pathologic stage (**D**), and pathologic T stage (**E**). (**F-I**) The post-progression survival curves (PPS; **F**), and distant metastasis-free survival curves (DMFS; **G**), and recurrence free survival curves (RFS; **H, I**) between the EZH2-high and EZH2-low expression groups of BRCA in Kaplan–Meier plotter databases. **P* < 0.05, ***P* < 0.01, ****P* < 0.001, *****P* < 0.0001.

**Fig. S2.** **DOX upregulates EZH2 expression**

(**A-D**) EZH2 mRNA levels in 231-WT cells exposed to DOX for 12 h (**A**), 24 h (**B**), 36 h (**C**), and 48 h (**D**). (**E-H**) EZH2 mRNA levels in 231-ADR cells exposed to DOX for 12  h (**E**), 24  h (**F**), 36  h (**G**), and 48  h (**H**). (**I-L**) Time‑course analysis of EZH2 mRNA in 231‑ADR cells treated with fixed DOX doses of 0.5 μM (**I**), 1 μM (**J**), 2 μM (**K**), and 3 μM (**L**) for 0, 12, 24, 36, and 48 h. All data were obtained from independent experiments (n = 3). **P* < 0.05, ***P* < 0.01, ****P* < 0.001, *****P* < 0.0001; ns, not significant.

**Fig. S3. Synergistic effects of EZH2 inhibition and DOX on BRCA cell viability**

(**A-D**) Dose response of DOX-induced cytotoxicity in 231-ADR (**A**) and MCF7-ADR (**B**) cells treated with or without GSK126 pretreatment (n = 4). (**C–D**) Dose–response of DOX-induced cytotoxicity in parental 231-WT (**C**) and MCF7-WT (**D**) cells with or without TAZ pretreatment (n = 4). (**E-G**) Synergy plot using ZIP (**E**), Bliss (**F**), and Loewe (**G**) models for 231-ADR cells treated with TAZ and DOX. (**H-K**) Synergy plot using ZIP (**H**), Bliss (**I**), Loewe (**J**), and HSA (**K**) models for 231-ADR cells treated with GSK126 and DOX. (**L**) Synergy analysis of TAZ and DOX in MCF7-ADR cells (HSA model). (**M–N**) Drug response matrices of 231-ADR cells treated with TAZ (**M**) or GSK126 (**N**) in combination with DOX. (**O**) Drug response matrix of MCF7-ADR cells treated with TAZ and DOX. **P* < 0.05, ***P* < 0.01, ****P* < 0.001, *****P* < 0.0001; ns, not significant.

**Fig. S4. EZH2 inhibition enhances DOX-induced DNA damage across BRCA models.**

(**A–B, D–E**) γH2AX **immunofluorescence** in 231-ADR and MCF7-ADR cells treated with EZH2 inhibitors plus DOX: representative images (**A–B**) and quantification (**D–E**) (n = 3). Scale bar: 20 μm. (**C**) Quantification of γH2AX fluorescence intensity corresponding to Fig. 4J (n = 3). (**F–I**) Quantification of γH2AX and EZH2 protein levels in 231-ADR cells (n = 3).

**Fig. S5. Targeting EZH2 suppresses DNA damage repair pathways**

(**A, B**) Intersection analysis of genes downregulated by DOX versus control and upregulated by TAZ+DOX, with KEGG (B) pathway enrichment analysis of the overlapping genes. (**C-D**) GO (**C**) and KEGG (**D**) pathway enrichment analysis of downregulated DEGs in the TAZ+DOX group compared with DOX alone. (**E-G**) GSEA comparing the combination treatment (TAZ + DOX) with DOX alone for KEGG pathways: base excision repair (**E**), mismatch repair (**F**), and nucleotide excision repair (**G**).

**Fig. S6. Antioxidant regulation in BRCA cells under EZH2 inhibition**

(**A–E**) qRT–PCR analysis of representative antioxidant-related genes in 231-ADR cells under the indicated treatments (n = 3). (**G–H**) Superoxide dismutase (SOD) activity in 231-ADR cells under the indicated treatments (n = 3). **P* < 0.05, ***P* < 0.01, ****P* < 0.001, *****P* < 0.0001; ns, not significant.

**Fig. S7. Biosafety evaluation *in vivo***

(**A**) Photography of isolated tumors. (**B-H**) Routine hematological analyses of mice after the administration of DOX, TAZ, TAZ+DOX, and Lip (n = 4).

**Fig. S8. Histopathological evaluation of organ toxicity *in vivo*.**

(**A**) Pathological H&E staining of major organs. Scale bar: 200 μm. (**B**) Masson staining of cardiac tissue under the indicated treatments. Scale bar: 200 μm.
