# Supplementary material for: EZH2 blockade reverses doxorubicin resistance by inducing metabolic vulnerability and enhancing DNA damage in breast cancer
Source: Front Pharmacol. 2026 May 14;17:1786648. doi: 10.3389/fphar.2026.1786648 (PMC13216663; doi:10.3389/fphar.2026.1786648)
Supplement: Supplementary file 3 [file Table1.docx]

| Table S1. Materials in this study. |  |  |
| --- | --- | --- |
| REAGENT or RESOURCE | **SOURCE** | **IDENTIFIER** |
| Antibodies |  |  |
| Anti-EZH2 antibody | Proteintech | Cat#21800-1-AP |
| Anti-γH2AX antibody | Zenbio | Cat#R381558 |
| Anti-Ki67 antibody | Abcam | Cat#ab16667 |
| beta-actin mouse monoclonal antibody | Proteintech | Cat#66009-1-Ig |
| HRP-conjugated Affinipure Goat Anti-Mouse IgG(H+L) | Proteintech | Cat#SA00001-1 |
| HRP-conjugated Goat Anti-Rabbit IgG(H+L) | Proteintech | Cat#SA00001-2 |
| Dylight 549, Goat Anti-Rabbit IgG | Abbkine | Cat#A23320 |
| Critical commercial assays |  |  |
| RIPA Buffer | Solarbio Science & Technology | Cat#S2100 |
| H&E Stain Kit | Solarbio Science & Technology | Cat#G1120 |
| BCA Protein Assay Kit | Solarbio Science & Technology | Cat#PC0020 |
| DAPI Solution | Solarbio Science & Technology | Cat#C0065 |
| Hoechst 33342 | Solarbio Science & Technology | Cat#C0030 |
| Masson’s Trichrome Stain Kit | Solarbio Science & Technology | Cat#G1346 |
| Calcein/PI Cell Viability/Cytotoxicity Assay Kit | Beyotime | Cat#C2015M |
| MMP assay kit with JC-1 | Beyotime | Cat#C2006 |
| Enhanced ATP Assay Kit | Beyotime | Cat#S0027 |
| Crystal Violet Staining Solution | Beyotime | Cat#C0121 |
| Total Superoxide Dismutase Assay Kit | Beyotime | Cat#S0101S |
| One-step TUNEL In Situ Apoptosis Kit | Elabscience | Cat# E-CK-A322 |
| DAB Kit | Zhongshan Goldenbridge Biotechnology | Cat#ZLI-9018 |
| Anti-mouse/rabbit IgG Polymer Detection System | Zhongshan Goldenbridge Biotechnology | Cat#PV-6000 |
| CCK-8 | APExBIO | Cat#K1018 |
| HiScript III RT SuperMix | Vazyme | Cat#R323 |
| ChamQ Blue Universal SYBR qPCR Master Mix | Vazyme | Cat#Q312-02 |
| Dihydroethidium (DHE) | MedChemExpress | Cat#HY-D0079 |
| 2',7'-Dichlorodihydrofluorescein Diacetate (DCFH-DA) | MedChemExpress | Cat#HY-D0940 |
| Trizol Reagent | Thermo Fisher Scientific | Cat#15596026CN |
| Chemicals |  |  |
| Tazemetostat | MedChemExpress | Cat#HY-13803 |
| GSK126 | MedChemExpress | Cat#HY-13470 |
| Sodium Carboxymethyl Cellulose | MedChemExpress | Cat#HY-Y1889A |
| Cholesteryl Hemisuccinate | Saitong | Cat#C11839 |
| DSPE-PEG2000 | Ponsure Biotechnology | Cat#K09115 |
| Leclthin | Aladdin Co. Ltd | Cat#L105732 |
| DMEM Basic(1X) | Gibco | Cat#C11995500BT |
| 4% Paraformaldehyde Fixative(4% PFA) | EallBio | Cat#02.11182 |
| Doxorubicin | Solarbio Science & Technology | Cat# D8740 |
| EDTA Antigen Retrieval Solution | Solarbio Science & Technology | Cat#C1034 |
| Sodium Citrate Antigen Retrieval Solution | Solarbio Science & Technology | Cat#C1032 |
| Fetal bovine serum (FBS) | ProCell | Cat#164210 |
| penicillin/streptomycin solution | EallBio | Cat#03.12001A |
| Triton X-100 | Beyotime | Cat#ST1723 |
| BSA | Aladdin Co. Ltd | Cat#B265994 |
| N, N-dimethylformamide | ACMEC Biochemical | Cat#N67025 |

Table S2. Sequence of primers used in this study.

| h-EZH2 | Forward | AATCAGAGTACATGCGACTGAGA |
| --- | --- | --- |
|  | Reverse | GCTGTATCCTTCGCTGTTTCC |
| h-ACTB | Forward | CATGTACGTTGCTATCCAGGC |
|  | Reverse | CTCCTTAATGTCACGCACGAT |
| BRCA1 | Forward | CTGAAGACTGCTCAGGGCTATC |
|  | Reverse | AGGGTAGCTGTTAGAAGGCTGG |
| BRCA2 | Forward | GGCTTCAAAAAGCACTCCAGATG |
|  | Reverse | GGATTCTGTATCTCTTGACGTTCC |
| ERCC1 | Forward | GCTGGCTAAGATGTGTATCCTGG |
|  | Reverse | ATCAGGAGGTCCGCTGGTTTTCTT |
| MCM2 | Forward | TGCCAGCATTGCTCCTTCCATC |
|  | Reverse | AAACTGCGACTTCGCTGTGCCA |
| MCM2 | Forward | TGCCAGCATTGCTCCTTCCATC |
|  | Reverse | AAACTGCGACTTCGCTGTGCCA |
| XRCC1 | Forward | CGGATGAGAACACGGACAGTGA |
|  | Reverse | GAAGGCTGTGACGTATCGGATG |
| POLB | Forward | TGCAGAGTCCAGTGGTGACATG |
|  | Reverse | ATGAACCTTTTGTAACTGCTCCAC |
| RPA2 | Forward | GAGCACCTATCAGCAATCCAGG |
|  | Reverse | CCTTCAGGTCTTGGACAAGCCT |
| PDHX | Forward | CAACTCCTGGACAACCCAATGC |
|  | Reverse | CTCCAAGGTCACAGTCAGCAGT |
| PDHA1 | Forward | GGATGGTGAACAGCAATCTTGCC |
|  | Reverse | TGTAACTGTGGAAGGAGGCTGG |
| PDHB | Forward | TGTAACTGTGGAAGGAGGCTGG |
|  | Reverse | CATCAGCACCAGTGACACGAAC |
| CS | Forward | CACAGGGTATCAGCCGAACCAA |
|  | Reverse | CCAATACCGCTGCCTTCTCTGT |
| DLD | Forward | GGGACTAGAAGAGCTGGGAATTG |
|  | Reverse | CATCCTCTGCTTTGTGAGCCAG |
| MDH1 | Forward | CGGTGTCCTAATGGAACTGCAAG |
|  | Reverse | CATCCAGGTCTTTGAAGGCAACG |
| CYP1A1 | Forward | GATTGAGCACTGTCAGGAGAAGC |
|  | Reverse | ATGAGGCTCCAGGAGATAGCAG |
| NRF2 | Forward | CACATCCAGTCAGAAACCAGTGG |
|  | Reverse | GGAATGTCTGCGCCAAAAGCTG |
| SOD1 | Forward | CTCACTCTCAGGAGACCATTGC |
|  | Reverse | CCACAAGCCAAACGACTTCCAG |
| SOD2 | Forward | CTGGACAAACCTCAGCCCTAAC |
|  | Reverse | AACCTGAGCCTTGGACACCAAC |
| CAT | Forward | GTGCGGAGATTCAACACTGCCA |
|  | Reverse | CGGCAATGTTCTCACACAGACG |

**Table S3. Summary of significantly enriched pathways identified by GSEA.**

| Term | ES | NES | pval | FDR | Group |
| --- | --- | --- | --- | --- | --- |
| DNA replication | -0.504 | -2.04 | p＜0.001 | 0.002 | TAZ+DOX vs Ctrl |
| Mismatch repair | -0.474 | -1.72 | 0.014 | 0.022 | TAZ+DOX vs Ctrl |
| Nucleotide excision repair | -0.427 | -2.0 | p＜0.001 | 0.002 | TAZ+DOX vs Ctrl |
| Base excision repair | -0.501 | -2.15 | p＜0.001 | 0.0 | TAZ+DOX vs Ctrl |
| Oxidative phosphorylation | -0.547 | -2.71 | p＜0.001 | FDR＜0.001 | TAZ vs Ctrl |
| Citrate cycle (TCA cycle) | -0.609 | -2.38 | p＜0.001 | FDR＜0.001 | TAZ vs Ctrl |
| Pyruvate metabolism | -0.426 | -1.76 | p＜0.001 | 0.012 | TAZ vs Ctrl |
| Mismatch repair | -0.560 | -1.76 | 0.005 | 0.011 | TAZ+DOX vs DOX |
| Nucleotide excision repair | -0.516 | -1.95 | p＜0.001 | 0.002 | TAZ+DOX vs DOX |
| Base excision repair | -0.536 | -1.91 | p＜0.001 | 0.003 | TAZ+DOX vs DOX |
